# Patient-centred care: a scoping review of measures of patient centredness in healthcare in low- and middle-income countries (LMICs)

**DOI:** 10.3332/ecancer.2026.2102

**Published:** 2026-03-27

**Authors:** Anna Cabanes, Cindy Sun, Diksha Thakkar, Carolyn Taylor, Phuong Thao Le

**Affiliations:** 1 Global Focus on Cancer, South Salem, NY 10590, USA; 2 Boston University School of Public Health, Boston University, Boston, MA 02215, USA

**Keywords:** person-centred care, patient-centred care, people-centred care, LMIC, indicator

## Abstract

Person- or patient centredness (PC) is increasingly recognized as a core component of high-quality healthcare and a critical pathway to achieving equitable, person-responsive cancer services. While the concept of PC has been widely adopted and measured in high-income countries, its implementation and evaluation in low- and middle-income countries (LMICs) remain limited and inconsistent. This scoping review maps existing research on measures of PC in LMICs, with a specific focus on cancer care. We identified 345 studies published between 2004 and 2022 that reported on PC in LMICs, of which 20 focused on cancer. Most studies were conducted in upper-middle-income countries and used cross-sectional designs. Patient satisfaction was the most commonly measured domain, followed by communication and shared decision-making. However, foundational aspects of PC – such as patient rights, coordination of care and culturally responsive services – were rarely evaluated. Only 41% of studies used validated tools, and most interventions occurred at the facility level, with few system-level evaluations. Using the Santana et al. framework, which categorizes PC domains into structure, process and outcomes, we found that structural and systemic components of PC were particularly underrepresented in LMIC research. This suggests a gap between policy ambitions and practical implementation, especially in cancer care settings where multidisciplinary coordination and patient engagement are essential. This review advances the field by systematically documenting how PC is currently measured in LMICs and identifying significant limitations in scope, equity and contextual relevance. Findings highlight the urgent need for validated, culturally adaptable tools and frameworks that reflect the realities of LMIC health systems. Addressing these gaps is essential for scaling person-centred cancer care and strengthening health systems toward universal health coverage.

## Background

People-centred, person-centred and patient-centred healthcare systems respect the experience, values, needs and preferences of people in the planning, coordination and delivery of care. People-centred care looks at communities, person-centred care looks at the whole individual and patient-centred care (PCC) looks at the person specifically as a patient receiving treatment.

The concept of patient-centredness (PC) is not new, but its implementation has primarily been concentrated in high-income countries (HICs) and has been shown to improve patient outcomes, optimize resource utilisation, reduce costs and enhance satisfaction with care [1]. In 2018, three major reports emphasized PC as a cornerstone for enhancing the quality of healthcare systems across all resource levels, an essential component of functional health systems and a vital mechanism for achieving equitable access to quality care through universal health coverage [2–4].

Despite the recent extensive body of research and the prominence of PC in policy discussions, the concept lacks a universally accepted definition. An analysis of existing theoretical frameworks reveals multiple models, each emphasising different dimensions of PCC, for example, patient-provider relationship and communication (COM), integration of medical and non-medical care, coordination and continuity of care, patient participation and involvement of family and friends, among others [5–9]. These are understood and applied differently across contexts and among diverse stakeholders, which impacts the effective implementation and evaluation of patient-centred initiatives.

Among the frameworks reviewed, the model proposed by Santana *et al* [8] stands out for its practical applicability. This framework integrates the components of PC into the Donabedian model for healthcare improvement [10], categorising them into structure, process and outcomes for practical implementation in healthcare systems.

**Figure 1.**
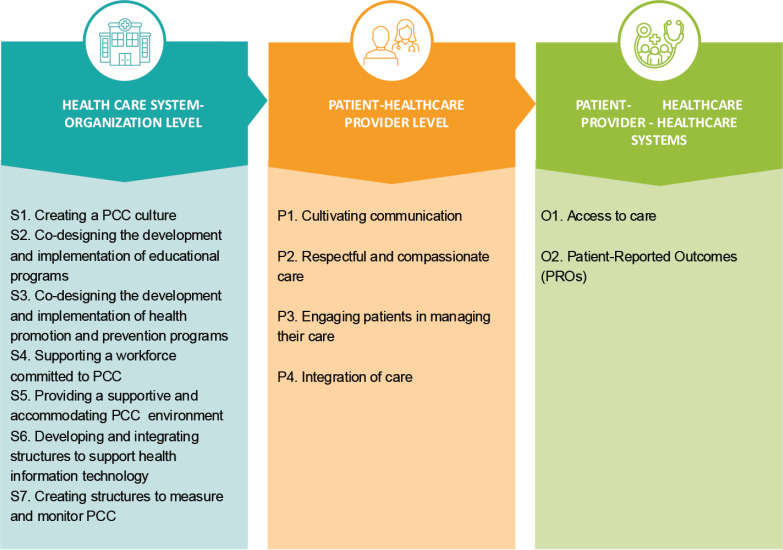
Conceptual framework for PCC adapted from Santana et al [8].

Figure 1 presents the conceptual framework adapted from Santana *et al* [8]. Structure domains encompass aspects of PCC at both foundational (healthcare system) and organisational levels. Process domains include four subdomains focused on the interactions between patients and healthcare providers. Outcome addresses two domains centred on patient experience (PE) and health outcomes. Measuring the impact of PC requires the use of appropriate indicators to assess its quality. By breaking PC into these distinct components, this framework provides a solid foundation for evaluation and quality improvement.

The challenge of measuring PC is reflected in the diverse array of tools developed, tested and applied across various settings [8, 11]. While some tools aim to comprehensively evaluate PC, many focus on specific aspects of the concept. Certain tools are designed to measure PC over time, while others assess it within the scope of a single healthcare visit. Additionally, some tools are designed for use within individual facilities, whereas others can be applied across entire healthcare systems.

The most commonly measured domains include patient perceptions of PC [12], patient-reported experiences [13], fostering a PC culture, physician-patient COM [6, 14–17] and shared decision making (SDM) [5], among others. However, the majority of these studies have been conducted in HICs.

In low- and middle-income countries (LMICs), there is growing recognition of the importance of PC, but significant challenges persist in assessing its impact [18–20]. While frameworks and tools for measuring PC are established in high-income settings, their applicability in LMICs is often limited by contextual differences. Existing tools may not adequately capture the unique sociocultural, economic and systemic factors that shape healthcare experiences in these regions. For example, variations in health literacy, resource constraints and distinct patient-provider dynamics often necessitate adaptation and validation of PC measurement instruments.

Efforts are required to develop and validate tools that accurately reflect the realities of LMIC healthcare systems. Context-specific research is essential to create standardized yet adaptable approaches for evaluating PC in these settings. Such tools should accommodate the challenges of limited resources while providing reliable and actionable insights to improve healthcare delivery.

This scoping review aimed to identify measures of PC used in LMIC contexts and analyse a valid set of indicators suitable for resource-constrained settings. While the scoping review was not limited to a specific disease type, we conducted a sub-analysis of articles focused on cancer, aligning with the primary focus of our work.

## Methods

### Search strategy

This study followed PRISMA guidelines for scoping reviews [21]. Keywords were developed by reviewing published literature for terms related to people-centred, people-focused, patient-centred and patient-focused care, as well as countries categorized as low income, lower-middle income or upper-middle income according to the 2022 World Bank classification (https://datatopics.worldbank.org/world-development-indicators/the-world-by-income-and-region.html).

We searched the following databases: PubMed, ERIC and CINAHL and restricted articles to those published in English between January 2004 and December 2022. Data collection and analysis were conducted from January through July 2023.

### Inclusion/exclusion criteria

Articles were included in this review if they were original research studies focused on measuring PCC or any of its domains and were implemented in LMICs. The review included articles across all disease and care settings within LMICs.

Articles were excluded from this review if they did not report indicators or measures, were conducted in a HIC, were published in a language other than English, lacked full-text availability or were conference abstracts. We excluded studies in which some components of PC were evaluated but not in relation to PC. For example, studies measuring satisfaction with surgical outcomes solely based on clinical results were not included. Table 1 illustrates the inclusion and exclusion criteria applied during the screening process.

### Data extraction and assessment of quality

The initial search retrieved 1,219 articles. After removing duplicates, 1,144 articles were selected for screening. Three research team members conducted a two-phase screening process for each selected article. Each article was reviewed by two people (DT, CS), with a third reviewer (AC) resolving disagreements through consensus. This process resulted in the exclusion of 547 articles. A subsequent full-text review resulted in the exclusion of an additional 252 articles for not specifically evaluating the interventions.

**Figure 2.**
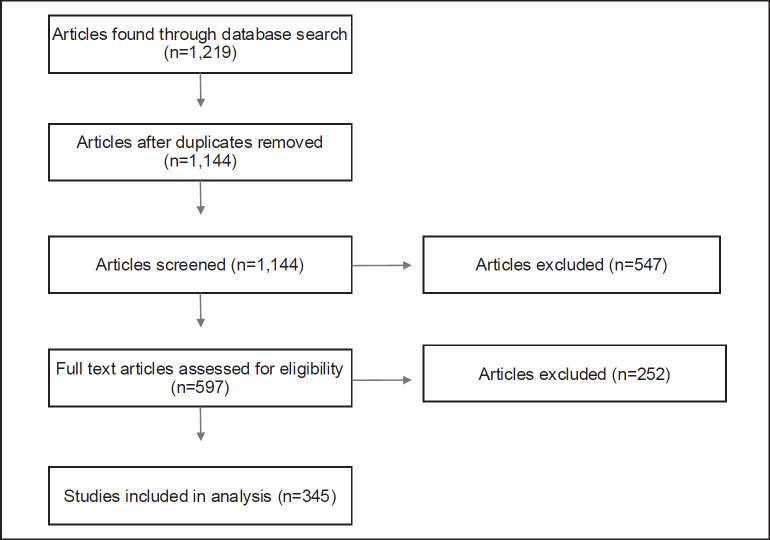
PRISMA flow diagram.

After screening, 345 articles were included in this review. Figure 2 presents the PRISMA flow diagram illustrating the selection process. Two members of the review team extracted key information from each article, including: title, lead author, year of publication, study country and resource level, whether the study was conducted in the general patient population or directed to a specific disease, intervention details, study outcome, healthcare system level; study design, whether there was a control group and the number of participants; the general domain of PC being measured; the tools used to measure it and whether the tool or instrument had been previously validated, was validated in the study or was not validated. We sought to extract all the fields listed above for each article. However, in some cases, studies did not report all the variables. We followed the Synthesis Without Meta-analysis reporting guidelines (https://swim.sphsu.gla.ac.uk/) for reporting of the results.

Note that, for clarity throughout the review, we use PC as the overarching concept and PCC as a specific subdomain for which measurement tools have been developed.

## Results

A total of 345 studies were included in the review, reporting measures of PC in LMICs. The number of articles on PC measures progressively increased from 2004 through 2022, peaking in 2021. Table 2 summarizes the characteristics of the studies included.

### Income levels of the countries in the included studies

Of the 345 selected studies, 191 (55%) articles were conducted in upper-middle income countries, 86 (25%) in LMICs and 61 (18%) in low-income countries (six studies were implemented in several countries with varying income levels). The countries with the most studies included were China (*n* = 72), Ethiopia (*n* = 37), Turkey (*n* = 24), Malaysia (*n* = 23) and Nigeria (*n* = 16). Table 3 shows the five countries of each income level group with more articles included, and the number of articles from each.

### Study design

293 studies used a cross-sectional study design, 6 utilized randomized control trial design and 3 were cohort studies. There was no control group in 323 studies, whereas 15 studies included a control group. The total number of participants in the studies varied from 17 to 83,046. Study subjects varied from nurses, physicians, patients, medical providers, to facilities and districts also surveyed.

Of the 6 RCT, only two studies targeted family/PC, 3 assessed satisfaction as a secondary outcome of a non-patient centred intervention, and one used quantitative data from a randomized control trial and offered descriptive analysis without an intervention [22].

## Population under study

Most studies focused on measuring PCC in the general patient population (*n* = 214), compared to other patient groups. After the general patient population, people living with cancer (*n* = 20), with tuberculosis (*n* = 19), and HIV/AIDS (*n* = 17) or attending maternal health services (*n* = 15) were most often analysed. Healthcare providers were included in 12 articles. Among the articles focused on cancer, breast, colorectal, hepatocellular, lung, liver and hematologic cancers were most frequently studied.

### Level/s of the healthcare system where the intervention was implemented

Of the studies reviewed, 302 implemented the intervention at the health facility level (one or more facilities), and 30 were conducted across the health system. Thirteen studies (*n* = 13) were categorized as ‘other’, including interventions implemented in pharmacies, dialysis centers or other healthcare-related facilities.

### Measuring different PC domains

The elements of PC most frequently evaluated were patient satisfaction (PS) (*n* = 223), PCC (*n* = 59), patient provider COM (*n* = 16) and PE (*n* = 8). Other key domains of PC, such as patients' rights (0%), quality of life (<5%), SDM (3.3%) and multidisciplinary care, were never or seldom measured.

### Measuring PC at the facility level

Most frequently, PC was measured at one facility or several facilities (*n* = 302). These facilities were primary level facilities (*n* = 83), and tertiary level or specialized care facilities (*n* = 90). Nineteen articles were implemented at secondary level facilities or general hospitals (GHs) (*n* = 84). Table 4 presents the distribution of specific PCC domains across different healthcare levels: primary, secondary, tertiary and GH/district.

PS was the most frequently analysed domain across all healthcare levels, followed by PCC. Quality of care (QoC) was assessed across all levels, with the highest proportion observed at the secondary care level (5.3%), while education (ED) was reported in both secondary (5.3%) and GH/District levels (3.6%), but not in other levels. As expected, SDM was primarily reported at the tertiary care level (3.3%), where more specialized and complex care decisions are typically made.

### Measuring PC across the healthcare system

Thirty studies assessed PCC across a healthcare system. Of these, a third (*n* = 10) used validated tools. Countries included in these studies are China, Kenya, Lebanon, Pakistan and Turkey. 

The validated tools used to assess PC included the European Patients Evaluate General/Family Practice PS survey [23, 24]; an adapted Chinese version of Hospital Consumer Assessment of Healthcare Providers and Systems [25], the World Health Organisation SAGE [26], SERVQUAL tool [27], the Person-centred Maternity Care Scale [28] and the Picker Patient Experience Questionnaire [29]. Additionally, two tools were validated during the studies; an adapted version of the Commonwealth Fund [30] and Harris Interactive Questionnaire [30] used in multiple countries, including Brazil, Colombia, Mexico and El Salvador; and the 1998 Kaiser National Household Survey [31], a client satisfaction tool validated in a study conducted in South Africa. Several other tools were used in health system-wide studies but were not validated.

### Measures of PC in cancer

Our scoping review was not limited to a specific disease type. More than half of the analysed articles measured PC in the general patient population. Among the 345 articles analysed, 20 focused on measuring PC in cancer. The search results indicated that studies mostly were not directed at a specific type of cancer but some included a mix of cancer types.

Most interventions originated from upper-middle income countries (*n* = 15), with the highest representation from China (*n* = 7), followed by Mexico (*n* = 5), Malaysia (*n* = 2) and Turkey (*n* = 2). Fifty percent of these studies measured PS, while others assessed patient-centred care,

QoC, SDM, doctor-patient relationships and COM, and patient ED. However, other domains and subdomains of cancer PC were not measured. Notably, seventy percent of the articles measuring PC in cancer utilized validated tools.

Certain domains of PC, such as PS and QoC, are commonly assessed using a variety of measurement tools. In some cases, these domains are evaluated with a single tool that captures specific aspects of the PE. However, more comprehensive evaluations may use composite measures that combine several scales to provide a broader understanding of the determinants of PS and care quality. These composite measures might integrate factors like COM effectiveness, emotional support, respect for patient preferences and coordination of care, offering a more nuanced view of how well healthcare services align with the principles of PC.

Among the 20 reviewed articles focused on cancer, 8 assessed PS using validated instruments. Four of these studies used generic measures, specifically the Patient Satisfaction Questionnaire short forms (PSQ-9 or PSQ-18) [29, 32–34], while the remaining four employed cancer-specific tools from the EORTC PATSAT family, including inpatient (IN-PATSAT32) and outpatient (OUT-PATSAT33/PATSAT-C33) versions or related adaptations [35–38]. Thus, PS in cancer care was measured using a mix of generic and oncology-specific instruments across studies.

## Discussion

In LMICs, significant gaps remain in the evaluation of PCC. Most studies in these settings focus on a limited range of measures, primarily assessing PS rather than the broader domains of PC. While several studies have examined PC within specific geographic regions, there is a lack of research identifying appropriate indicators and instruments tailored to regional healthcare contexts. To the best of our knowledge, our study is the first to assess measures of PC across countries of different income levels.

For instance, a systematic literature review by Alkhaibari *et al* [18] identified key elements of PC in the Middle East and North Africa, and suggests that PC is only partially practiced and supported in the region, with cultural factors significantly influencing its implementation. This review emphasizes the need for a regionally relevant definition of PC that incorporates cultural nuances and underscores the importance of considering these cultural dynamics when designing and implementing PC strategies.

Similarly, a scoping review exploring PC in Latin America revealed considerable variation across countries in their emphasis on PC versus family-centred care (FCC): among the 32 studies analysed, nearly half were conducted in Brazil. The review found that patient information was the most highlighted dimension of PC, while physical support received the least emphasis [20].

In sub-Saharan Africa, a study identified seven key themes of PC: patient provider COM, understanding, respect, compassion, stigmatisation, decision-making and trust [19]. This review highlighted significant challenges such as poor COM and mistreatment in patient-provider interactions, recommending targeted training programmes for healthcare providers to enhance PC practices.

The three studies share a common focus on PC within specific geographic regions and underscore the need for culturally tailored approaches to PC implementation. Despite regional differences, all studies stress the lack of standardized indicators for evaluating PC and emphasize the need for frameworks that address cultural and systemic nuances.

To analyse our scoping review, we adopted Santana's conceptual framework which offers a structured roadmap for guiding healthcare systems and organisations in delivering PC [8]. This framework applies the Donabedian model to characterize PC domains into three main groups: structure, process and outcome. Structure domains pertain to the healthcare system or the context in which care is provided, forming the foundation for PC. Process domains focus on the interactions between patients and healthcare providers, while outcome domains reflect the tangible results of PC, demonstrating its value through measurable impacts such as PS and experience.

Most of the articles we reviewed primarily focus on measuring single aspects/dimensions of PC, such as PS and PE. However, effective PC implementation requires attention to the structural aspects of healthcare systems, such as integrating PC principles into professional ED and fostering meaningful partnerships with patients and patient organisations. It also necessitates strong process elements that enhance patient-provider interactions. Despite their importance, our review found that these critical structural and process domains are rarely measured in LMICs, highlighting a significant gap in the evaluation and implementation of PC in these settings.

Additionally, we found that PS and PE are measured inconsistently across studies and settings. In some cases, PS is assessed as a single variable using a straightforward yes/no scale or a numerical scale. While this approach provides a quick snapshot of satisfaction, it may fail to capture the nuances of PEs, such as how well their specific needs were addressed or how they perceived COM with their provider.

In contrast, other studies measure PS as a composite variable encompassing multiple dimensions of care. These dimensions may include COM quality, timeliness of services, respect for patient preferences, physical comfort and emotional support.

Across the six studies that reported a RCT design, the ones reporting person- and family-centred approaches generally improved patient or caregiver experience, and, in several cases, strengthened clinical outcomes, with the strongest effects seen in interventions designed to enhance COM or family involvement [42, 43]. The studies focused on patient-centred COM in Uganda and family centred neonatal care in China, demonstrated clear, statistically significant gains, while simple behavioural nudges like reminder calls also improved the quality of the intervention and satisfaction [44]. In contrast, large system or policy changes resulted in more variable effects on satisfaction [45, 46]. This underscored that patient-centred benefits may be better assessed in studies explicitly designed to strengthen patient/FCC rather than as a secondary or downstream outcome of a non–patient-centred intervention.

Regarding the measurement of patient-centred cancer care, we found a limited focus on cancer patients. While many studies measured PC in general patient populations, only 20 articles specifically examined PC in oncology-targeted cancer patients. This highlights a significant gap in the literature, suggesting that the unique needs and challenges of implementing PC in cancer care remain underexplored and may require greater attention in future research.

Most interventions measuring PC in cancer originated from upper-middle income countries, possibly reflecting emerging healthcare reforms or targeted investments in quality of oncology care in these economies. A notable proportion (70%) of studies used validated tools, which is encouraging for the reliability of their findings. However, the limited scope of domains assessed, and the variability of instruments used, suggests that existing tools may not comprehensively capture all elements of PC relevant to cancer care.

For example, most studies used validated PS instruments, supporting the credibility and internal validity of the findings. However, the split between generic PSQ tools and cancer-specific EORTC PATSAT measures introduces heterogeneity that limits direct comparability across studies and complicates synthesis. While PSQ instruments offer feasibility and cross-setting comparability, the EORTC PATSAT scales provide greater sensitivity to oncology-specific care processes and setting-specific experiences. This variability highlights an ongoing tension between feasibility and specificity in PE measurement and underscores the need for greater alignment around core patient-centred metrics in cancer research. For policymakers and implementers, this trade-off highlights the need to balance how easy it is to compare results and the effort required to collect data with the level of detail needed to improve cancer services.

## Conclusion

In summary, our findings reveal significant gaps in the literature regarding PC in LMICs, particularly in terms of the comprehensiveness of assessed domains and the geographic distribution of studies. Future research should aim to broaden the scope of PC evaluations to include underrepresented domains and settings and expand studies to low-income countries where data remains limited. Additionally, developing or adapting validated measurement tools tailored to specific healthcare settings could provide more robust insights into the delivery and impact of PC in these settings.

This study provides a snapshot of the use of standardized measures of PCC in LMICs, using Santana’s framework of structure, process, and outcome domains. To the best of our knowledge, our study is the first to assess measures of PC across countries of different income levels. However, a key limitation of the study is the heterogenous use of the term ‘PCC’, which led to a retrieval of a wide variety of scales and tools, making it challenging to draw clear conclusions. Future research should focus on specific aspects of PCC, particularly foundational domains or disease-specific dimensions, to enhance the precision and applicability of measurement approaches.

## List of abbreviations

CHLT, Cancer health literacy test; COM, Communication; ED, Education; EORTC, European Organisation for Research and Treatment of Cancer; FCC, Family-centred care; GH, General hospital; HICs, High-income countries; IN-PATSAT32, In-patient satisfaction with care questionnaire [32-item scale developed by EORTC]; LIC, Low-income country; LMICs, Low- and middle-income countries; PCC, Patient-centred care; PE, Patient experience; PPOS, Patient-Practitioner Orientation Scale; PRISMA, Preferred Reporting Items for Systematic Reviews and Meta-Analyses; PSQ-18, Patient satisfaction questionnaire [18-item short form]; QoC, Quality of care; SDM, Shared decision-making; SERVQUAL, Service quality [an instrument to measure service quality]; UMIC, Upper-middle income country; WHO SAGE, World Health Organisation Study on Global AGEing and Adult Health.

## Conflicts of interest

The authors have no conflicts of interest to declare that are relevant to the content of this article.

## Funding

The authors did not receive support from any organisation for the submitted work.

## Informed consent

Not applicable.

## Author contributions

AC, CT, PTL developed the concept and design. AC, CS and DT worked in acquisition of data and reviewed the included articles. All authors made substantial analysis and interpretation of data; drafted and revised the article for important intellectual content; and have seen and approved the final version of the manuscript.

## Figures and Tables

**Table 1.** Inclusion and exclusion criteria.

	Inclusion criteria	Exclusion criteria
Setting	Low, lower-middle and upper-middle income countries	HICs
Intervention	PCC, PCC initiatives reporting an indicator or a measure	Studies reporting needs but not reporting a PCC intervention or indicator
Setting	All levels of healthcare settings	Interventions implemented outside of the healthcare settings
Study design	Original research articles of all study designs	Opinion pieces, commentaries, reviews, protocols with no published results, dissertations, news articles
Language, date of publication	English language, published after 2004	Languages other than English, published before 2004

**Table 2.** Characteristics of studies included in the scoping review by country income level.

		*N*	%	LIC	LMIC	UMIC	MIX
Studies included	# studies	345	100.0	61	86	191	6
Type of study	Cross-sectional	293	84.9	52	74	163	4
	Randomized study	6	1.7	2	1	3	0
	Other	46	13.3	7	11	25	2
Subdomain	PS	223	64.6	41	61	119	2
	PCC	59	17.1	11	15	31	2
	ED	5	1.4	1	0	3	1
	Doctor-patient relationship	5	1.4	0	2	3	0
	PE	8	2.3	1	1	6	0
	COM	16	4.6	2	3	11	0
	SDM	4	1.2	0	0	4	0
	QoC	11	3.2	2	4	6	1
	Doctor-patient relationship	5	1.4	0	2	3	0
Population studied	General	214	62.0	39	52	120	4
	People living with cancer	20	5.8	2	1	17	0
	Healthcare providers	12	3.5	0	1	11	0
	Tuberculosis	19	5.5	4	7	7	1
	Psychological	9	2.6	2	1	4	0
	HIV	17	4.9	2	4	1	1
	Reproductive	4	1.15	1	3	1	0
	Maternity	15	4.3	7	5	3	0
	Other	12	3.5	4	8	27	0
Health system level	One facility+	302	87.5	57	75	168	2
	Across HC system	30	8.7	3	8	18	1
	None	13	3.8	1	2	4	3
Income level	UMIC	191	55.4	-	-	-	
	LMIC	86	24.9	-	-	-	
	LIC	61	17.7	-	-	-	
	Mix	7	2.0	-	-	-	
Validated instruments	Yes	142	41.2	24	36	80	2
	No	99	28.7	15	22	59	3
	Validation study	69	20.0	17	16	36	0

**Table 3.** Number of articles in the top five countries per income level group.

		Upper-middle income (*n* = 191)	Lower-middle income (*n* = 86)	Low income (*n* = 61)
Five top countries in number or articles included, by income level	1	China (*n* = 72)	Nigeria (*n* = 16)	Ethiopia (*n* = 37)
	2	Turkey (*n* = 24)	Pakistan (*n* = 15)	Uganda (*n* = 8)
	3	Malaysia (*n* = 23)	Nepal (*n* = 8)	Rwanda (*n* = 4)
	4	Brazil (*n* = 13)	Vietnam (*n* = 7)	Afghanistan (*n* = 4)
	5	South Africa (*n* = 12)	Tanzania (*n* = 5)	Malawi (*n* = 2)

**Table 4.** Specific PC domains addressed in the included articles across different healthcare levels.


	Primary	Secondary	Tertiary	GH/ district
PS	57 (68.7%)	15 (78.9%)	62 (68.9%)	56 (66.7%)
PCC	14 (16.9%)	3 (15.8%)	12 (13.3%)	13 (15.5%)
COM	4 (4.8%)	0	3 (3.3%)	3 (3.6%)
PE	3 (3.6%)	0	0	0
QoC	3 (3.6%)	1 (5.3%)	3 (3.3%)	4 (4.8%)
ED	0	1 (5.3%)	0	3 (3.6%)
SDM	0	0	3 (3.3%)	0
	83 (100%)	19 (100%)	90 (100%)	84 (100%)

**Table 5.** Most common validated tools used to assess PC in cancer.

Tool	Domain measured	Country	Validated	# articles that use the tool	Reference
Multi item questionnaire: [1, 2, 4] measures the health-related quality of life EORTC IN-PATSAT3215	Determinants of PS	Bulgaria	Individual scales validated elsewhere, not validated in Bulgaria	1	[35]
Health information wants questionnaire (HIWQ- 21 items)	Health information wanted from doctors and nurses [Comms]	China	No, adapted from a validated scale	1	[39]
Patient-centred quality of cancer care questionnaire (PCQCCQ)	Quality of PC Scale encom-passes five domains: [1] timely care, [2] clarity of information, [3] information for treatment decision-making, [4] care to address biopsychosocial needs, and [5] respectful and coordinated care.	Mexico	Yes, in Mexico	5	Doubova 2021 Doubova 2020 [experiences] Doubova 2020 [validation study] Doubova 2020 Doubova 2021 [Doubova *et al* 2021; Doubova, Terreros-Muñoz *et al* 2020; Doubova and Pérez-Cuevas, 2020; Doubova and Pérez-Cuevas 2021] [38]
Patient-Practitioner Orientation Scale (PPOS)	Patient-provider relationship and PS	Malaysia	Yes	2	[32, 40]
Short -Form Patient Satisfaction Questionnaire (PSQ-18)	PS	Malaysia	Yes	1	[41]
Cancer Health Literacy Test [CHLT]	Cancer health literacy	Malaysia	No	1	[40]
Chinese Patient Satisfaction Questionnaire-9 items (ChPSQ-9)	PS	China	Yes	3	[29, 34]
IN-PATSAT32 (EORTC in-patient satisfaction with care questionnaire -Sinhala version)	PS	Sri Lanka	Yes	1	[36]
EORTC PATSAT-C33	PS	Ethiopia	Yes	1	[37]
